# Hypoxia and DNA-Repair Radiosensitivity Signatures Are Associated with Radiotherapy-Modified Survival in TCGA Breast Cancer, with External Prognostic Validation of the Hypoxia Score in METABRIC

**DOI:** 10.3390/biotech15020028

**Published:** 2026-03-31

**Authors:** Jimmy Carter Osei, Mei-Han Chen, Tim A. D. Smith

**Affiliations:** 1 School of Computer Science and Electronic Engineering, Bangor University, Bangor LL57 1UT, UK; jms24xfh@bangor.ac.uk (J.C.O.); meiheyo80@gmail.com (M.-H.C.); 2 Nuclear Futures Institute, Bangor University, Bangor LL57 1UT, UK

**Keywords:** breast cancer, radiotherapy, hypoxia, DNA damage repair, radiosensitivity, TCGA

## Abstract

Radiotherapy (RT) is one of the main treatments for breast cancer, but response varies between patients. Tumour hypoxia and intrinsic radiosensitivity are major determinants of response to RT. Using TCGA-BRCA, a 563-gene hypoxia meta-signature was built by combining curated hypoxia gene sets from MSigDB with published hypoxia metagenes (Buffa, Winter, Elvidge, Fardin, and related sets). After Cox screening and penalised regression, a simple three-gene hypoxia score (CP, GPC3, STC1) was derived. In parallel, based on DSB-repair factors highlighted by Mladenov et al. as key regulators of intrinsic radiosensitivity, a four-gene radiosensitivity (RS) signature (ATR, RPA2, BLM, MRE11A) was trained using only RT-treated patients. In TCGA, both signatures were prognostic and showed significant interaction with RT status in Cox models. The hypoxia score was strongly associated with worse outcomes in RT-untreated patients, but this effect was much weaker in RT-treated patients (Hypoxia × RT HR = 0.009, *p* = 0.044). The RS score showed a similarly strong interaction with RT (RS × RT HR = 0.011, *p* = 0.003). When we combined both signatures into one interaction model, it gave the best performance (C-index = 0.785), and both interaction terms stayed independently significant. The hypoxia score was then validated externally in METABRIC (N = 1979; 1143 events), where it remained associated with overall survival, although more weakly than in TCGA (HR = 1.34, 95% CI: 1.10–1.63; *p* = 0.0042). Overall, these results suggest that hypoxia and DSB-repair capacity capture two complementary sides of radiosensitivity and RT-modified survival patterns, and they support further prospective testing and validation in independent datasets with strong RT annotation.

## 1. Introduction

Radiotherapy (RT) is a key part of breast cancer management, helping to reduce local recurrence and improve long-term outcomes across many stages of disease [1,2]. Even though RT works well at the population level, benefit varies between patients. People with similar clinical features may have very different outcomes, likely because of differences in tumour biology, the tumour microenvironment, and other treatments given alongside RT [3]. Therefore, there is a real need for biologically informed methods to predict patient treatment benefit, identifying patients who are likely to respond well to RT and those requiring a more intensive approach or a different treatment regimen.

Breast cancer is a heterogeneous disease entity that includes carcinomas and sarcomas. Sarcomas represent <1% of breast cancers and are a biologically distinct mesenchymal tumour type, which exhibit different clinical and radiobiological behaviour to carcinomas. Radiation-associated breast angiosarcoma, for example, represents a distinct secondary malignancy after breast-conserving treatment [4]. Accordingly, the present analysis was restricted to primary breast carcinoma cases represented within TCGA-BRCA.

Two major biological drivers of radiosensitivity have been recognised: tumour hypoxia (low oxygen levels) and intrinsic radiosensitivity. Hypoxia reduces the oxygen-mediated chemical fixation of radiation-induced DNA radical damage, thereby increasing the probability that some lesions remain chemically reversible and reducing effective radiosensitivity. Further, hypoxia induces activation of programmes that support angiogenesis, epithelial–mesenchymal transition, metabolic changes, and treatment resistance [5,6,7]. Many hypoxia-related gene-expression signatures have been proposed across cancer types, including well-known metagenes from large pan-cancer studies [8].

RT kills cells mainly by inducing DNA double-strand breaks (DSBs), which are repaired by pathways such as homologous recombination or non-homologous end joining [9,10]. Important regulators like ATM, ATR, TP53BP1, BRCA1, BLM, RPA1/2, and MRN-complex components are central to recognising damage and coordinating repair, thereby influencing intrinsic radiosensitivity [11]. Many existing radiosensitivity scores (including RSI-style approaches and breast RT gene signatures) have mainly been used for prognosis or recurrence-risk stratification, rather than explicitly testing RT interaction effects in survival models [12,13].

Hypoxia captures microenvironmental impacts and modified transcriptomics that can reduce effective radiation damage, while DSB-repair capacity captures intrinsic cellular ability to detect and repair DNA lesions, two different but potentially complementary mechanisms of radioresistance. Datasets such as TCGA-BRCA enable linking transcriptomics with treatment annotations and survival outcomes at scale [14]. Importantly, Cox model interaction terms allow differentiation between purely prognostic and biomarkers predictive of treatment response [15]. Recent reviews have also highlighted growing interest in clinical biomarkers of tumour radiosensitivity, radiogenomic stratification, and radiosensitisation strategies in breast cancer, reinforcing the need for clinically interpretable molecular models that can distinguish prognostic effects from RT-modifying effects [16,17].

In this study, mechanistically curated gene sets are combined with penalised regression and interaction-focused survival modelling to test how hypoxia and DSB-repair-based radiosensitivity relate to overall survival in TCGA breast cancer, including formal interaction tests with RT status. To check whether the hypoxia axis generalises beyond TCGA, the derived hypoxia score is also examined in the independent METABRIC cohort as an external prognostic validation dataset. We hypothesised that (i) each biological axis would show independent RT interaction effects in TCGA, and (ii) a combined model using both axes would discriminate better than either signature alone.

## 2. Materials and Methods

### 2.1. Overall Study Design

**Figure 1:**
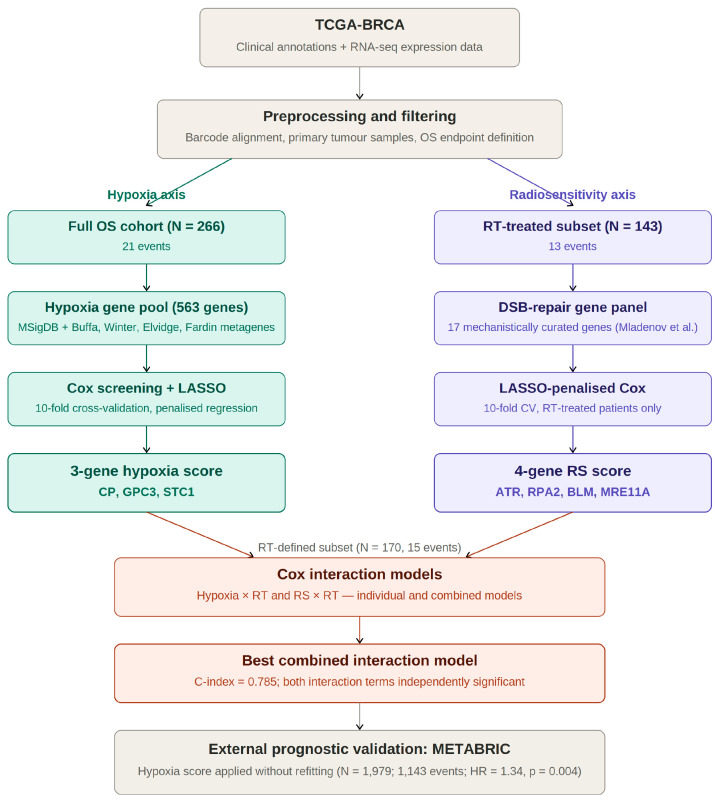
Schematic overview of the study workflow. A hypoxia signature (left branch, teal) was derived from a 563-gene pool in the full OS cohort, while a radiosensitivity (RS) signature (right branch, purple) was derived from 17 DSB-repair genes in RT-treated patients only. Both scores were then tested in Cox interaction models within the RT-defined subset (N = 170) and the hypoxia score was externally validated in METABRIC [11].

A retrospective transcriptomic re-analysis of TCGA-BRCA [14] was undertaken to test whether tumour hypoxia (microenvironmental) and DNA double-strand break (DSB) repair capacity (intrinsic) gene expression signatures can either individually or in combination most robustly predict overall survival (OS) and response of breast carcinoma patients to radiotherapy (RT). The workflow had three main steps (Figure 1): (i) building a hypoxia expression score from a mechanistically grounded hypoxia gene pool; (ii) building a radiosensitivity (RS) score using curated DNA repair factors linked to DSB processing and intrinsic radiosensitivity; and (iii) testing whether RT status modifies the association between each signature and OS using Cox models with signature × RT interaction terms, run for each score separately and then again in a combined model. All analyses were done using de-identified public data and were limited to primary breast tumour samples.

### 2.2. Data Acquisition and Preprocessing

#### 2.2.1. TCGA-BRCA Clinical and Expression Data

Clinical annotations and RNA-seq expression data for TCGA-BRCA, shown in Table 1, were pulled from the Genomic Data Commons (GDC) [18] and FireBrowse [19], in line with the original TCGA breast cancer characterisation paper [14]. We downloaded the clinical data in XML-derived tabular format, so the nested TCGA structure was kept. Gene-expression data were taken as gene-level matrices (RSEM normalised; log_2(x + 1) transformed), with samples indexed using full TCGA barcodes.

To align identifiers across files, we truncated each sample barcode (e.g., TCGA-A1-A0SB-01A-11R-A144-07) to the first 12 characters (e.g., TCGA-A1-A0SB), which matches the patient-level IDs used in the clinical tables. If a patient had multiple aliquots, expression values were averaged across aliquots. The dataset was restricted to primary tumour samples only (sample type code “01”). Histological subtype information, where available in the clinical annotation, was used for cohort description. Radiotherapy-related metadata were queried across all available radiation fields, including radiation type, dosage, and start/end timing variables; however, completeness of these fields was limited and inconsistent across patients, so detailed dose- and timing-resolved modelling was not feasible.

In the TCGA-BRCA cohort, the primary tumour specimens are generally obtained at initial diagnosis or surgery rather than after radiotherapy. Therefore, the analysed transcriptomic data were interpreted as reflecting primary tumour biology prior to RT exposure. Because TCGA does not provide a fully harmonised specimen-level variable linking RNA-seq procurement time directly to RT timing for every case, we cannot formally confirm pre-RT sampling for every individual patient; however, the study was designed as a primary tumour expression analysis rather than a post-treatment tissue study.

#### 2.2.2. Inclusion and Exclusion Criteria

Patients were included if they had: (i) RNA-seq data from a primary tumour; (ii) enough clinical follow-up to calculate overall survival (OS) time and vital status; and (iii) good mapping of expression identifiers to HGNC gene symbols for most of the candidate genes. Patients were excluded if OS time was missing or zero; if follow-up was under one month with unclear event status; or if the expression and clinical IDs could not be matched properly. After filtering and merging the clinical and expression tables, 266 patients were kept for the OS analysis.

RT status could only be determined confidently for a subset of patients, using a composite RT indicator (described below). RT information was clear for 170 patients: 143 had evidence of RT, and 27 had no evidence of RT. The remaining patients had uncertain RT status and were removed from RT-specific analyses. Because of this, all RT interaction models and the RS derivation were done in the 170-patient RT-defined subset, while the initial hypoxia modelling used the full 266-patient OS cohort.

#### 2.2.3. Survival Endpoints

Overall survival was defined as the time from initial pathological diagnosis to death from any cause, or to the last follow-up date if the patient was still alive. OS time in days was taken from patient.days_to_death for patients who died, and from patient.days_to_last_followup or patient.days_to_last_known_alive for censored patients. When more than one follow-up field was available, the most recent non-missing value was used. OS time was then converted to months by dividing by 30.44. Patients with negative or clearly unrealistic times (suggesting data-entry artefacts) were excluded.

Vital status was taken from the relevant TCGA clinical field (e.g., patient.vital_status) and recoded into a binary event variable (1 = death, 0 = censored). If vital status was missing or inconsistent, other follow-up fields were checked; any cases still non-resolvable were excluded.

#### 2.2.4. Extraction and Harmonisation of Radiotherapy Status

RT-related information in the TCGA clinical XML is spread across several nested sections, including baseline patient fields, follow-up modules, and new tumour event records. All radiation-related fields that could reasonably reflect RT exposure were queried, including:

- patient.radiation_therapy;
- patient.radiations.radiation.radiation_type,.radiation_dosage,.days_to_radiation_therapy_start/end;
- patient.follow_ups.follow_up.radiation_therapy and the corresponding radiation fields across follow-up entries;
- new tumour event modules with additional_radiation_therapy flags.

Because recording practices differed across contributing centres, a composite RT indicator (RT) was applied to limit misclassification. RT was set at 1 if any of the queried fields suggested RT (for example, a “YES” entry, an explicit radiation course, or a non-zero dose value). RT was set at 0 only when all available radiation-related fields consistently indicated no RT (for example, explicit “NO” entries) and the clinical record looked sufficiently complete overall. If different modules disagreed, the patient was classified conservatively as RT-treated. Patients with completely missing radiation information were excluded from RT-specific analyses.

Additional radiotherapy metadata were extracted from the TCGA clinical XML, including radiation type, recorded dosage, dose units, fraction number, and days to RT start and end where available. Among the 170 RT-defined patients, radiation type was available for 150, radiation dosage for 141, days to RT start for 146, and days to RT end for 147. Dose units were recorded mainly in cGy (132 cases), with a smaller number in Gy (13 cases), and were missing in 25 cases. After harmonising available dose units to Gy, the recorded dose distribution had a median of 60 Gy (IQR 50.4–64 Gy), but with substantial heterogeneity and several extreme values, indicating that the TCGA radiation fields likely mix differing course structures or non-standardised annotations. Overall, 141/170 patients (82.9%) had both usable dose and timing information available, 9/170 (5.3%) had timing information only, and 20/170 (11.8%) had neither usable dose nor timing data. These RT metadata were therefore used descriptively to characterise treatment annotation rather than as harmonised quantitative covariates in the survival models.

### 2.3. Gene Expression Processing

#### 2.3.1. Mapping to Gene Symbols and Filtering

The expression matrices were mapped to HGNC gene symbols using TCGA-provided annotation tables. If multiple identifiers mapped to the same gene symbol, they were averaged to get one expression value per gene per patient. Any identifiers that could not be mapped were dropped.

To limit noise from genes that do not really vary or are not meaningfully expressed, a variance-based filter was applied across the expression matrix and removed genes with near-zero variance or very low expression in most samples. After this filtering step, the global expression matrix for the 266-patient OS cohort contained about 20,500 genes. Hypoxia modelling and score derivation were done in this 266-patient expression space. For the later interaction analyses, we then subset the same matrix to the 170 RT-defined patients, without refitting the hypoxia model.

#### 2.3.2. Normalisation and Scaling

The RSEM normalisation and the $\log_{2}$(x + 1) transformation were kept exactly as provided by TCGA/FireBrowse [20]. No extra between-sample normalisation was applied because the cohort had already been processed using a harmonised pipeline. For model building, expression values for the candidate genes were standardised to mean zero and unit variance within the cohort used for derivation:

$$z_{gi}=\frac{E_{gi}-\mu_{g}}{\sigma_{g}},$$

where $E_{gi}$ is the $\log_{2}$(x + 1) expression of gene g in patient i, and $\mu_{g}$ and $\sigma_{g}$ are the mean and standard deviation of gene g across patients in the relevant derivation cohort (the 266-patient cohort for hypoxia, and the RT-treated subset for RS training). The same scaling parameters were used when calculating scores in the 170-patient RT-defined subset.

To check whether any large batch structure could be confounding the survival results, a principal component analysis (PCA) on the global expression matrix was run, and the top components, coloured by RT status, tumour subtype, and key clinical covariates, were examined. No obvious dominant batch separation was identified, so an explicit batch correction was not applied.

### 2.4. Hypoxia Signature Derivation

#### 2.4.1. Hypoxia Gene Pool and Univariable Screening

A 563-gene hypoxia meta-signature was built by bringing together hypoxia-related gene sets from the MSigDB Hallmark and C2 collections [21], including HALLMARK_HYPOXIA and other curated hypoxia pathways, and combining these with published hypoxia metagenes such as Buffa [8], Winter [22], Elvidge [23], and Fardin [24], plus related signatures described in hypoxia meta-analyses [5,8]. The union of all gene symbols was taken, and duplicates were removed to get the final 563-gene pool.

Within the TCGA-BRCA OS cohort (N = 266), each gene was screened using a univariable Cox model. Genes that showed an adverse association with survival (HR > 1) were then taken forward into penalised multivariable modelling.

#### 2.4.2. Penalised Multivariable Cox Model (LASSO)

The screened genes were then put into a LASSO-penalised Cox regression model [25] using glmnet. A ten-fold cross-validation was used to choose the penalty parameter λ. The final model was selected at λ_min and produced a simple three-gene hypoxia score, with non-zero coefficients for CP, GPC3, and STC1. The coefficient signs as fitted were kept, so that higher scores reflect higher predicted risk.

#### 2.4.3. Computation and Centring of Hypoxia Score

For each patient i, the hypoxia score was calculated as a weighted sum of z-scored gene expression:

$${HypoxiaScore}_{i}=\sum_{g\in H}\beta_{g}\;z_{gi},$$

where H=CP, GPC3, STC1 and $\beta_{g}$ are the fitted model coefficients. To make the interaction terms easier to interpret in later models, the score was centred within the analysis cohort:

$${\mathrm{Hypoxia}}_{c}(i)={HypoxiaScore}_{i}-\frac{1}{N}\sum_{j=1}^{N}{HypoxiaScore}_{j}.$$

The same three-gene model was used, along with the same coefficient weights for all downstream TCGA analyses, including when scoring patients in the RT-defined subset.

### 2.5. Radiosensitivity Signature Derivation

#### 2.5.1. Mechanistic DNA Repair Candidate Set

Radiosensitivity candidate genes were selected from DNA damage response and repair factors highlighted by Mladenov et al. for their roles in DSB recognition, end resection, checkpoint signalling, and homologous recombination [11]. The candidate list included ATM, ATR, PRKDC (DNA-PKcs), RBBP8 (CtIP), EXO1, RAD51, TP53BP1, BRCA1, RIF1, RPA1, RPA2, BLM, RTEL1, PCNA, and the MRN-complex genes MRE11A, NBN, and RAD50. After matching this list to the TCGA-BRCA expression matrix, expression values were available for 17 genes.

#### 2.5.2. Training Restricted to Radiotherapy-Treated Patients

To make sure the RS signature was capturing differences in outcome within the RT-treated group, the penalised Cox model was trained only in patients who received RT (RT = 1; N = 143, 13 events). The candidate genes were z-scored within this RT-treated subset, then a LASSO Cox model [25] was fitted using glmnet with 10-fold cross-validation. The idea here was to see which combination of the curated DSB-repair genes best explains variation in OS among patients who actually had RT.

After cross-validation, the model retained four genes with non-zero coefficients: ATR, RPA2, BLM, and MRE11A.

#### 2.5.3. Direction Correction and Score Computation

In the RT-only model, the genes that were selected had negative coefficients, meaning higher expression was linked to a lower estimated hazard among patients who received RT. But since we wanted the RS score to read as a “radioresistance” axis (i.e., higher score = higher expected risk in the RT setting), we flipped the sign of the linear predictor:

$${RSScore}_{i}=-\sum_{g\in R}{\hat{\beta }}_{g}z_{gi},$$

where R={ATR, RPA2, BLM, MRE11A} and ${\hat{\beta }}_{g}$ are the fitted coefficients. The RS score across was then centred on the 170-patient RT-defined subset:

$${\mathrm{RS}}_{c}(i)={RSScore}_{i}-\frac{1}{N_{RT}}\sum_{j=1}^{N_{RT}}{RSScore}_{j}.$$

Patients were excluded from the RS analysis if they were missing expression for any of the four genes. The score distribution was also checked for outliers and any major departures from an approximately normal shape.

### 2.6. Survival Modelling, Interaction Analysis and Discrimination

#### 2.6.1. Univariable and Interaction Cox Models

The association of each score with overall survival (OS) was tested using continuous Cox models [15]:

$$h(t|X)=h_{0}(t)exp\{\gamma_{1}\cdot {\mathrm{Score}}_{c}\},$$

where ${\mathrm{Score}}_{c}$ was either ${\mathrm{Hypoxia}}_{c}$ or ${\mathrm{RS}}_{c}$. To check whether the effect of a score changed depending on RT status, interaction models were fitted:

$$h(t|X)=h_{0}(t)exp\{\gamma_{1}\cdot {\mathrm{Score}}_{c}+\gamma_{2}\cdot \mathrm{RT}+\gamma_{3}\cdot ({\mathrm{Score}}_{c}\times \mathrm{RT})\}.$$

With this setup, $\gamma_{1}$ is the score effect in RT-untreated patients (RT = 0), and $\gamma_{3}$ indicates how much that effect is modified in RT-treated patients.

A combined model that included both centred scores and both interaction terms was fitted:

$$h(t|X)=h_{0}(t)exp\{\gamma_{H}\cdot {\mathrm{Hypoxia}}_{c}+\gamma_{R}\cdot {\mathrm{RS}}_{c}+\gamma_{RT}\cdot \mathrm{RT}+\gamma_{H\times RT}({\mathrm{Hypoxia}}_{c}\times \mathrm{RT})+\gamma_{R\times RT}({\mathrm{RS}}_{c}\times \mathrm{RT})\}.$$

Hazard ratios (HRs), 95% confidence intervals, and Wald *p*-values were reported, and nested models were compared using likelihood ratio tests.

#### 2.6.2. Assessment of Proportional Hazards and Diagnostics

The proportional hazards assumption was checked using scaled Schoenfeld residuals and the formal tests in cox.zph [26,27]. Residual plots for the hypoxia, RS, and RT terms were also studied to see if there were any clear patterns that would suggest non-proportional hazards. Martingale and deviance residuals were used to identify potentially influential observations, and sensitivity checks where possible outliers were excluded did not meaningfully change the conclusions.

#### 2.6.3. Discrimination and Performance Metrics

Discrimination was summarized using Harrell’s concordance index (C-index) [28], calculated with the concordance function in the survival package. Differences in C-index between models were treated carefully because the number of events was relatively small. Time-dependent ROC curves and AUC estimates were explored [29] at 36 and 60 months using timeROC as a complementary way to look at performance, without doing any time-specific optimisation.

#### 2.6.4. Risk Stratification and Kaplan–Meier Analysis

For descriptive plots, we split the hypoxia and RS scores at the cohort median to define “high” and “low” groups. Kaplan–Meier curves [30,31] were generated for (i) RT-treated patients stratified by hypoxia or RS; (ii) four-group comparisons combining RT status with high vs. low hypoxia; and (iii) the same type of four-group comparisons for RS. Log-rank tests were used to compare groups. These plots were meant to support the main results, not replace the primary interaction modelling.

### 2.7. Statistical Software and Reproducibility

All analyses were done in R (version 4.5.3) [32]. Survival modelling was run using the *survival* package [33], and penalised Cox regression was done with *glmnet* [34]. Kaplan–Meier plots were produced with *survminer*. Time-dependent ROC analyses were done using *timeROC* [29]. For data handling, we mainly used *data.table* and *dplyr* from the tidyverse ecosystem [35], and figures were generated with *ggplot2* [36].

A fully scripted R pipeline was used for reproducibility, with clear steps for data acquisition, clinical XML parsing, expression harmonisation, score computation, model fitting, diagnostic checks, and figure generation. The analysis scripts and score definitions will be made publicly available when the paper is published.

### 2.8. External Prognostic Validation in METABRIC

To see if the hypoxia axis generalises beyond TCGA, an external prognostic validation was done in the METABRIC cohort. Gene-expression data and clinical annotations were obtained from the METABRIC release and matched by patient identifier. Expression values were mapped to HGNC symbols and combined where needed so there was one value per gene.

The TCGA-derived hypoxia signature was applied without refitting the model. Specifically, CP, GPC3, and STC1 were z-scored within METABRIC, and the hypoxia score was computed using the fixed TCGA coefficients. Since TCGA and METABRIC use different platforms and have different scaling, within-cohort standardisation was used so the comparison is on a relative scale. In other words, this validation mainly tests whether the score can rank patients by risk consistently, rather than matching absolute risk levels.

Overall survival time (months) and event status were taken from METABRIC clinical fields (vital status and follow-up time), and records with missing or non-positive survival times were removed. The prognostic performance was evaluated in METABRIC using a continuous Cox model with the hypoxia score as the only predictor, and the results were summarised with HR, C-index, and time-dependent AUC at 60 months using timeROC [29]. Because METABRIC does not provide RT exposure in a way that is directly comparable to TCGA, RT interaction analyses were not run there; METABRIC was used specifically to validate the prognostic signal of the hypoxia score.

## 3. Results

### 3.1. Cohort Characteristics

The final OS cohort comprised 266 patients with primary breast tumours and 21 deaths and are shown in Table 2. Histologically, the cohort was dominated by carcinoma subtypes, principally infiltrating ductal carcinoma (187/266, 70.3%) and infiltrating lobular carcinoma (50/266, 18.8%), with the remaining 29 cases (10.9%) classified as other carcinoma types. Pathologic stage was centred mainly in stage IIA (90/266, 33.8%) and stage IIB (66/266, 24.8%), with additional stage IIIA representation (41/266, 15.4%). RT status could be confidently defined for 170 patients, of whom 143 had evidence of RT, and 27 had no evidence of RT.

### 3.2. Association of Hypoxia and RS Scores with Tumour Stage

Because tumour stage could potentially confound transcriptomic risk patterns, we examined whether the hypoxia and RS scores were associated with pathologic stage in TCGA-BRCA. Exploratory analyses showed that the hypoxia score varied significantly across detailed pathologic stage categories (Kruskal–Wallis χ^2^ = 17.13, df = 8, *p* = 0.0288), whereas the RS score did not show a statistically significant stage association (Kruskal–Wallis χ^2^ = 13.19, df = 8, *p* = 0.1056). These findings suggest that the hypoxia axis may partly track stage-related disease severity, while the RS axis appears less clearly explained by pathologic stage alone. However, the modest sample size, uneven distribution across stage strata, and limited event count mean that these analyses should be interpreted as exploratory rather than as definitive evidence of stage independence.

### 3.3. Hypoxia Signature Is Prognostic and Exhibits a Strong Radiotherapy Interaction

Most genes in the curated 563-gene hypoxia meta-signature were available in the TCGA-BRCA expression matrix used for overall survival (OS) analysis (N = 266). Starting with univariable Cox screening across the hypoxia gene pool, the genes with an adverse association (HR > 1) were taken into penalised multivariable modelling. Using a cross-validated LASSO Cox model at λ_min, we derived a simple three-gene hypoxia score made up of CP, GPC3, and STC1. After z-scoring each gene, the hypoxia score was right-skewed, but there were no extreme outliers.

In the full OS cohort (21 events), the continuous hypoxia score was strongly linked to poorer survival (HR per unit = 4.83, 95% CI: 2.55–9.15; *p* = 1.35 × 10^−6^), with a C-index of 0.70. This matches what we would generally expect based on the known relationship between hypoxia biology and more aggressive tumour behaviour.

**Figure 2:**
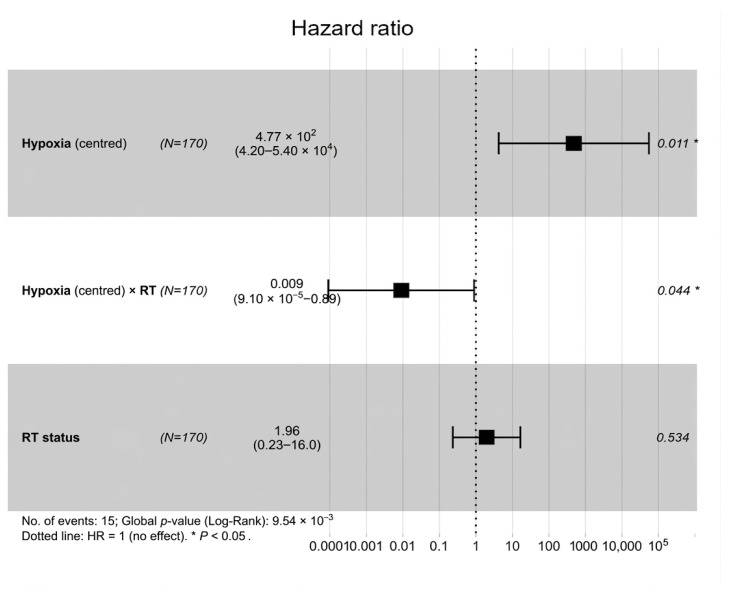
Cox model for centred hypoxia score (Hypoxia_c_), radiotherapy (RT) and their interaction. Squares denote hazard ratios and horizontal bars 95% confidence intervals. The vertical dotted line marks hazard ratio = 1. * *p* < 0.05.

**Figure 3:**
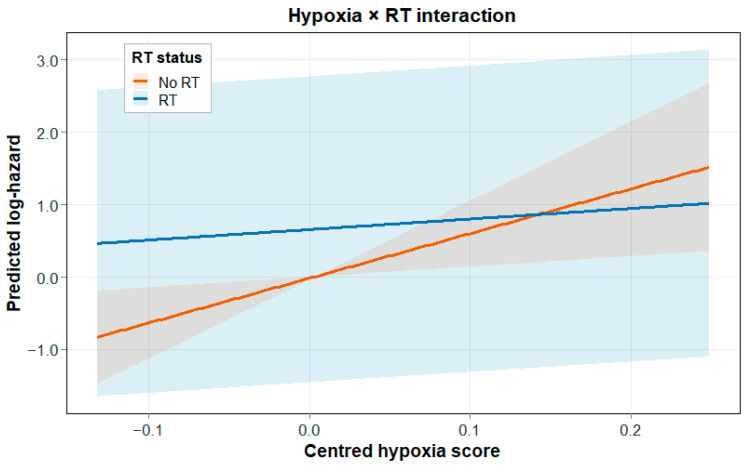
Predicted log-hazard as a function of Hypoxia_c_, stratified by RT status. Shaded ribbons denote 95% confidence intervals. The steeper slope in non-RT patients illustrates the Hypoxia × RT interaction.

To test whether the hypoxia effect differed depending on RT status, a Cox interaction model was applied in the RT-defined subset (N = 170, 15 events) (Figure 2). In this model, centred hypoxia (Hypoxia_c) showed a strong adverse association in RT-untreated patients (HR per unit = 477.3, 95% CI: 4.19–5.44 × 10^4^; *p* = 0.0107). Importantly, the Hypoxia_c × RT interaction term was strongly protective (HR = 0.0090, 95% CI: 9.09 × 10^−5^–0.885; *p* = 0.0442), which suggests that the hypoxia-associated hazard was much weaker in patients who received RT. This is also seen in the interaction plot, shown in Figure 3, where the predicted log-hazard increases much more sharply with Hypoxia_c in RT-untreated patients than in RT-treated patients.

Because there were very few events in the non-RT subgroup, the exact size of the subgroup hazard ratios needs to be treated carefully. The extremely large point estimate for Hypoxia_c in RT = 0 is likely driven by sparse events and the scaling/centring of the score. So the main point here is the direction of the effects and the fact that the interaction is statistically significant, supported by consistent Kaplan–Meier patterns, rather than the exact HR value in the RT = 0 group.

The Kaplan–Meier results matched the interaction model. In the four-group split (RT vs. No RT by High vs. Low hypoxia using the median), the worst survival was in the No RT/High hypoxia group, and RT was linked to better survival, especially among the high-hypoxia patients. Overall, these results support that hypoxia is prognostic in TCGA-BRCA and that its association with OS differs by RT status, consistent with hypoxia acting as a modifier of RT-associated survival patterns.

**Figure 4:**
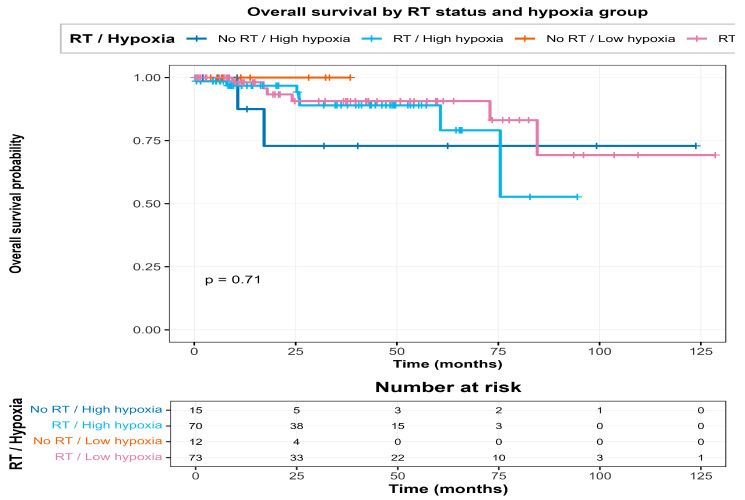
Kaplan–Meier curves for overall survival stratified by RT status and hypoxia group (high vs. low). The poorest outcomes are observed in the No RT/High hypoxia group.

Kaplan–Meier curves stratified by RT status and hypoxia group (high vs. low, median split) are shown in Figure 4, where the poorest survival was observed in the No RT/High hypoxia group, consistent with the significant interaction.

### 3.4. Radiosensitivity Signature Predicts Radiotherapy-Modified Hazard

To build a radiosensitivity (RS) signature based on DNA double-strand break (DSB) repair biology, a panel of 17 mechanistically curated DNA damage response and repair factors highlighted by Mladenov et al. as key determinants of cellular response to ionising radiation was curated [11]. All 17 candidates were available in the TCGA-BRCA expression matrix. Since the goal of RS was to capture variation in hazard within the radiotherapy (RT) setting, the model was trained only in patients with clear RT exposure (RT = 1; N = 143, 13 events). This way, the optimisation targets survival differences among treated patients, rather than picking up prognostic signals that might be driven by who was selected to receive RT.

**Figure 5:**
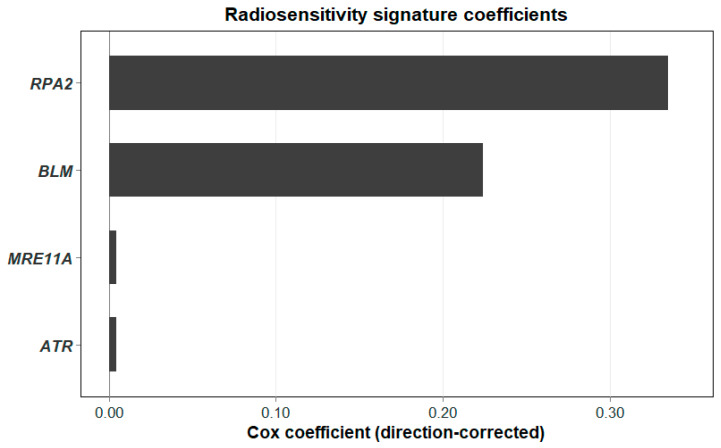
Direction-corrected Cox coefficients for the four-gene radiosensitivity (RS) signature (ATR, MRE11A, BLM, RPA2), rescaled so that higher scores correspond to greater radioresistance.

Using gene-wise z-scored expression values in the RT-treated subset, we fitted a LASSO-penalised Cox model with 10-fold cross-validation [34]. After cross-validation, the model retained four genes with non-zero coefficients: ATR, RPA2, BLM, and MRE11A. All four coefficients were negative in the RT-only fit, which is consistent with their known roles in checkpoint signalling, replication stress control, end resection, and early DSB sensing/processing [37,38,39]. For easier interpretation as a “radioresistance” axis, the sign of the linear predictor was flipped (multiplied by −1) so that higher RS values correspond to higher predicted hazard in RT-treated patients and are shown in Figure 5.

Within RT-treated patients, the original (non-inverted) linear predictor was strongly associated with OS (HR per unit = 0.062, 95% CI: 0.011–0.35; *p* = 0.0016), which is expected given the negative coefficients. After sign inversion, this corresponds to an approximate HR of 1/0.062 ≈ 16 per unit increase in the RS score, meaning higher RS reflects higher estimated hazard under RT. Discrimination within the RT-treated subset was good (C-index = 0.71) despite the small number of events. That said, because only 13 deaths occurred in the RT-trained subgroup, this RS model should be treated as exploratory, with more weight placed on direction and risk ranking than on the exact size of the effect estimates.

The RS association was then tested to determine whether it differed by RT status by applying the score across the full RT-defined cohort (N = 170) and fitting an interaction model [15,40,41]. In this model, centred RS (RS_c) showed a positive but non-significant trend in RT-untreated patients (HR per unit = 5.30, 95% CI: 0.55–50.72; *p* = 0.148). In contrast, the RS_c × RT interaction term was strongly protective (HR = 0.0109, 95% CI: 5.82 × 10^−4^–0.2047; *p* = 0.0025), suggesting the relationship between RS and hazard differs clearly between RT-treated and RT-untreated patients.

This is illustrated in the interaction plot, where the fitted log-hazard pattern with RS_c separates strongly by RT status. Kaplan–Meier curves in RT-treated patients split by the median RS score also support the direction-corrected score, with higher RS generally linking to poorer survival. The four-group RS × RT split shows the same overall structure; the No RT/High RS group has the worst outcomes, while RT is linked with better survival, particularly among patients with higher RS scores, consistent with RS-dependent modification of RT-associated hazard patterns.

**Figure 6:**
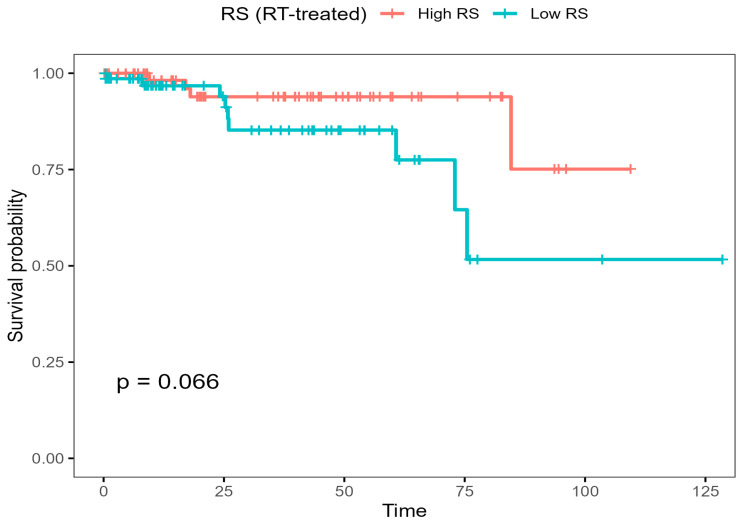
Kaplan–Meier curves for overall survival in radiotherapy-treated TCGA-BRCA patients, stratified by median RS score (high vs. low).

In KM analysis, shown in Figure 6, restricted to RT-treated patients, stratified by median RS score, patients with high RS tended to have worse OS (log-rank P≈0.066), consistent with the Cox interaction model.

**Figure 7:**
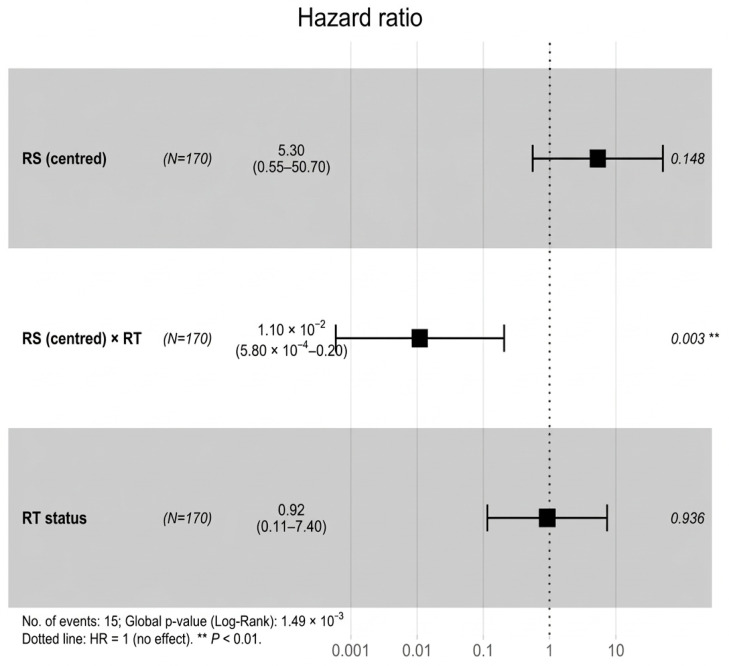
Cox model for centred RS score (RS_c_), radiotherapy and their interaction. The significant RS_c_ × RT term indicates that the prognostic effect of RS differs by RT status. The squares denote hazard ratios and horizontal; bars denote 95% confidence intervals. The vertical dotted line marks hazard ratio = 1. ** *p* < 0.01.

**Figure 8:**
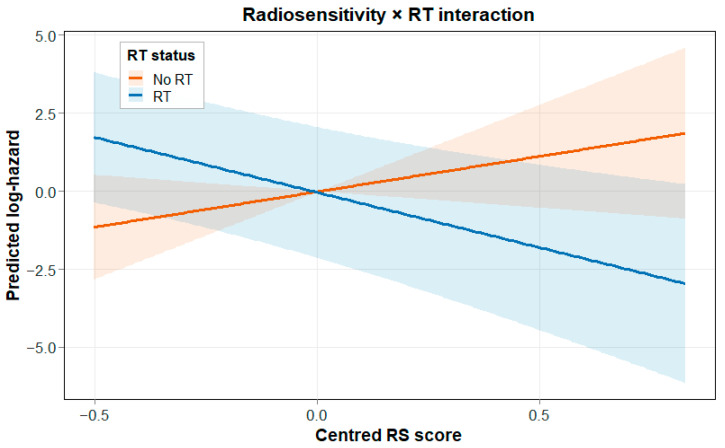
Predicted log-hazard as a function of RS_c_, stratified by RT status. Shaded ribbons denote 95% confidence intervals and illustrate the RS × RT interaction.

**Figure 9:**
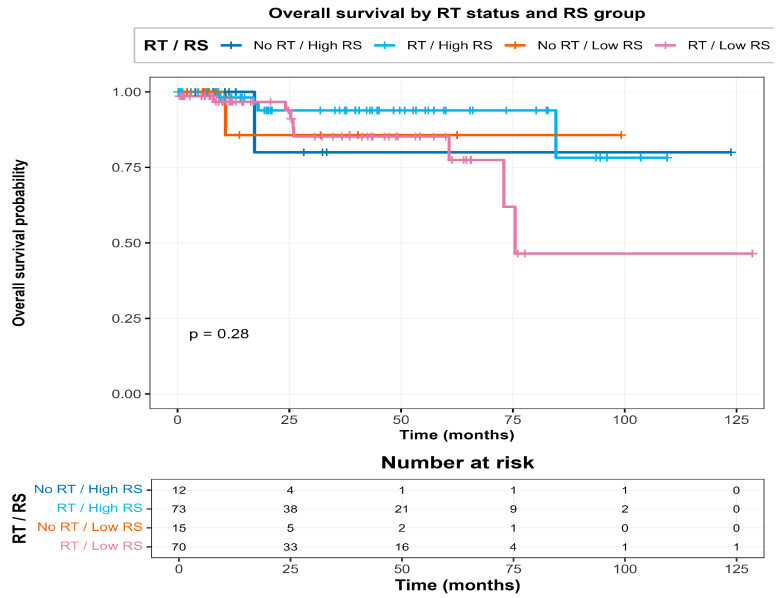
Kaplan–Meier curves for overall survival stratified by RT status and RS group (high vs. low). The poorest survival is observed in the no RT/high RS group.

Further inspection of the RS × RT interaction is shown in Figure 7, Figure 8 and Figure 9. The forest plot summarises the model, the interaction plot visualises the divergence in log-hazard by RT status across the RS score distribution, and the four-group KM plot illustrates combined stratification by RT and RS group.

### 3.5. Combined Hypoxia and Radiosensitivity Interactions Yield the Strongest Predictive Model

To check if hypoxia and DNA-repair-linked radiosensitivity are picking up different (non-overlapping) parts of outcome heterogeneity around radiotherapy (RT), a combined Cox model was fitted that included Hypoxia_c, RS_c, RT, and both score-by-RT interaction terms [15,40,41]. This setup tests effect modification for each biological axis while adjusting for the other one, enabling direct assessment of whether both signatures add independent information about RT-modified hazard patterns.

The combined interaction model gave the best discrimination out of all models we tested (C-index = 0.785) [42]. This was higher than the hypoxia-only (C-index = 0.64) and RS-only (C-index = 0.698) interaction models, which supports the idea that hypoxia and RS are capturing different sources of variation in outcomes. Likelihood ratio testing was consistent with this: the combined model fit significantly better than the single-interaction alternatives (χ^2^ = 28.28, df = 5, *p* = 3 × 10^−5^).

In the combined model, both interaction terms stayed independently significant. The Hypoxia_c × RT term remained strongly protective (HR = 1.83 × 10^−7^, 95% CI: 6.20 × 10^−14^–0.538; *p* = 0.0412), meaning the adverse hypoxia–survival relationship seen in RT-untreated patients was still greatly weakened in RT-treated patients even after accounting for RS. The RS_c × RT interaction also stayed significant (HR = 4.96 × 10^−4^, 95% CI: 2.16 × 10^−6^–0.114; *p* = 0.0061), showing an RT-dependent effect of the RS axis that persists after adjusting for hypoxia. Just like in the single-signature models, the small number of events likely makes these interaction HRs look extreme, so the main takeaway is strong directional evidence for effect modification rather than stable effect sizes.

Both Hypoxia_c and RS_c also kept positive main effects when RT was absent, with each linking to higher hazard among RT-untreated patients. This supports a model where microenvironmental low oxygen and intrinsic DNA repair capacity contribute separate components of radioresistance [5,9,43,44]. In practical terms, the interaction pattern is consistent with a “double contingency”: tumours that are both highly hypoxic and high on the RS axis have the worst outcomes without RT, but show the strongest reduction in risk when RT is given.

**Figure 10:**
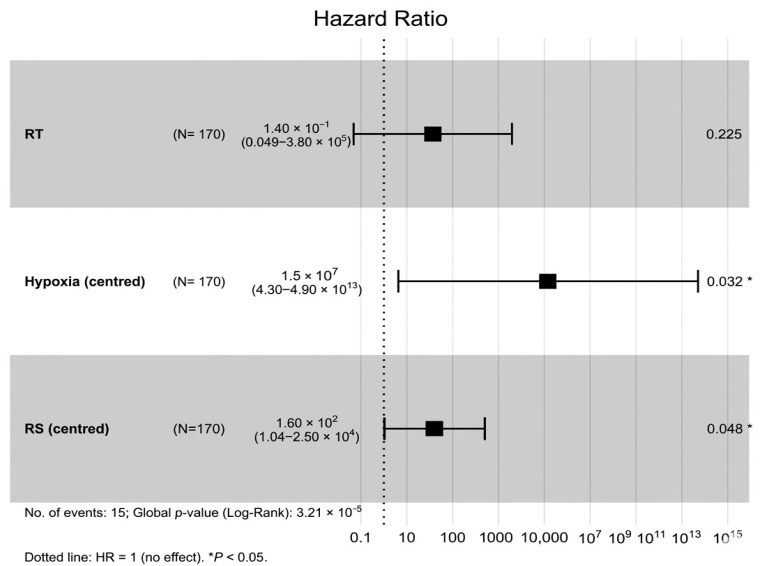
Combined Cox model including RT, Hypoxia_c_, RS_c_ and both interaction terms. Both Hypoxia × RT and RS × RT remain independently significant, indicating distinct predictive axes of radiotherapy response. Squares denote hazard ratios and horizontal bars denote 95% confidence intervals. The vertical dotted line marks hazard ratio = 1. * *p* < 0.05.

The combined forest plot (Figure 10) summarises the multivariable effects and shows the independent contribution of both interaction pathways. Overall, these results support a multi-axis picture where hypoxia-related microenvironmental stress and DSB repair-linked intrinsic biology work together to shape RT-modified survival patterns.

### 3.6. External Validation in METABRIC Supports a Retained Prognostic Hypoxia Signal

To check whether the hypoxia score generalises beyond TCGA, the fixed three-gene model (CP, GPC3, STC1) was applied to METABRIC (N = 1979; 1143 events) without refitting. Each gene was z-scored within METABRIC and then combined using the TCGA-derived coefficient weights to produce a continuous hypoxia score. This means the external test mainly evaluates whether the score preserves risk ranking across cohorts, rather than matching absolute risk levels.

In METABRIC, the hypoxia score was still significantly associated with overall survival, although the effect size was smaller than in TCGA (HR = 1.34, 95% CI: 1.10–1.63; *p* = 0.0042). Discrimination was modest (C-index = 0.521), with a 60-month time-dependent AUC of 0.55. Overall, these results support a reproducible prognostic signal for the hypoxia axis that transfers across cohorts, while also showing the expected change in performance when moving between datasets that differ in platform, patient mix, treatment patterns, and even how endpoints are defined.

### 3.7. Kaplan–Meier Analyses Highlight Distinct Patterns of Radiotherapy Benefit Across Hypoxia and RS Stratifications

To complement the continuous Cox models and the interaction analyses, Kaplan–Meier (KM) survival curves were plotted by splitting each signature at the cohort median. Even though median splits throw away information compared with keeping the scores continuous (so they can reduce statistical power), KM curves still give a straightforward visual view of the same directionality suggested by the interaction terms [30,45].

#### 3.7.1. Hypoxia-Stratified Survival Patterns

Among patients who received RT, hypoxia only separated the High and Low groups a little, which matches the Cox interaction result showing that the hypoxia–hazard relationship is weaker in the RT group. But in the No RT subgroup, patients with High hypoxia had much poorer survival, so the gap between High and Low risk groups was a lot clearer.

The four-group KM plot (RT vs. No RT × High vs. Low hypoxia) shows the same pattern clearly:

- No RT/High hypoxia had the worst overall survival.
- No RT/Low hypoxia was in the middle.
- RT/High hypoxia did better than the matching untreated High hypoxia group.
- RT/Low hypoxia had the best survival overall.

Overall, these curves give an easy visual confirmation of the Hypoxia_c × RT interaction; the survival disadvantage linked to hypoxia is mainly seen in untreated patients, and it is reduced in patients who received radiotherapy.

#### 3.7.2. Radiosensitivity-Stratified Survival Patterns

The RS signature showed a similar overall idea, but through a different biological mechanism. Among RT-treated patients, High RS was linked to poorer survival compared with Low RS, which fits with the direction-corrected RS score (higher RS = higher risk under RT). The four-group RS × RT split makes the pattern even clearer: the No RT/High RS group had the worst outcomes, while RT-treated patients with similar RS levels showed a visibly better survival trend.

Compared with hypoxia, the RS split looked more balanced between the High and Low groups, but the separation by RT status was stronger. This aligns with RS reflecting intrinsic DDR-linked biology that matters for radiation response, while hypoxia reflects microenvironmental limits that drive radioresistance through oxygen-dependent damage fixation and related transcriptional programmes [9,10,43].

#### 3.7.3. Comparison of Hypoxia and RS KM Stratifications

Overall, the KM curves point to four main takeaways:

- The risk linked to hypoxia shows up most clearly in patients who did not receive RT;
- The risk linked to RS is most obvious in the RT-treated group;
- Across the plots, the worst survival is consistently seen in high-score patients who did not receive RT;
- The visible benefit of RT looks strongest in the high-score subgroups.

These patterns match what we see in the Cox interaction models, supporting the idea that the signatures act as biologically sensible, clinically relevant modifiers of RT-associated survival patterns, not just general prognostic markers.

### 3.8. Association of Hypoxia and Radiosensitivity Scores with Pathologic Stage

Because tumour stage could potentially confound transcriptomic risk patterns, we examined whether the derived hypoxia and radiosensitivity (RS) scores were associated with pathologic stage in TCGA-BRCA. Using Kruskal–Wallis testing across available pathologic stage categories, the hypoxia score showed a significant association with stage (*p* = 0.0288), whereas the RS score did not (*p* = 0.1056). These findings suggest that part of the hypoxia signal may overlap with stage-related tumour severity, while the RS score appears less clearly stage-dependent in this cohort. Given the modest sample size and limited number of events, these analyses should be interpreted as exploratory.

## 4. Discussion

Radiotherapy is a cornerstone of treatment for many tumour types, including breast cancer. However, benefit from radiotherapy is non-uniform across patient populations [12], highlighting the need for predictive biomarkers of radiosensitivity that can guide treatment individualisation. Molecular analysis of tumour tissue has identified gene expression signatures with this potential [12], and there is growing interest in translating such genomic tools into clinical practice. Because response to radiotherapy is shaped by multiple biological variables, including tumour oxygenation and intrinsic DNA-repair capacity, we hypothesised that a combined model incorporating both hypoxia and radiosensitivity axes would provide stronger discrimination of radiotherapy-modified survival than either alone.

This study looks at two different biological drivers of radiosensitivity, tumour hypoxia and intrinsic DNA double-strand break (DSB) repair proficiency and shows that both are linked to radiotherapy (RT)-modified survival patterns in TCGA breast cancer. Even though there is strong experimental evidence that both processes affect radiation response, they are not often tested together using proper treatment–biomarker interaction modelling in a clinically annotated genomic cohort. By combining curated gene pools, penalised regression, and interaction-focused Cox analyses, we show that hypoxia and a DSB-repair-linked radiosensitivity (RS) signature add complementary information, and together they give the best discrimination in the RT-defined subset.

The hypoxia results fit well with what is already known from radiobiology. Oxygen helps “fix” radiation-induced DNA damage, so hypoxic parts of tumours are classically more radioresistant [6,43,46,47]. Oxygen is understood to enhance the chemical fixation of radiation-induced DNA radical lesions, helping convert transient radiation damage into persistent, biologically consequential damage. Under hypoxic conditions, this process is reduced, which contributes to radioresistance alongside broader hypoxia-associated transcriptional programmes. In this study, higher hypoxia scores were strongly linked to worse overall survival in patients who did not receive RT, but this relationship was weaker in RT-treated patients, which showed up as a significant Hypoxia × RT interaction. This needs to be interpreted carefully because the number of events is limited, but the pattern is still biologically plausible. RT could partly offset hypoxia-linked aggressiveness through cytoreduction even if some hypoxic subclones remain resistant; fractionated RT can allow some re-oxygenation between doses, and hypoxia-associated transcriptional programmes may behave differently once strong exogenous DSB stress is introduced.

The RS signature seems to capture a different, complementary axis tied to DNA damage response (DDR) and replication-stress biology. ATR, BLM, RPA2, and MRE11A are involved in checkpoint signalling, stabilising stressed replication forks, end resection, and early DSB sensing/processing, and together they connect directly to DSB recognition and homologous recombination-related biology [9,10,44].

At the same time, DDR gene expression is not a clean one-to-one measure of “repair proficiency.” It can also reflect proliferation, baseline replication stress, and genomic instability. Because of that, RS should be interpreted carefully and ideally backed up using cohorts with better RT detail and, where possible, independent functional readouts. Still, the RS model was trained only in RT-treated patients on purpose, so the fact that these DDR-linked genes were selected is at least consistent with DDR-related variation being informative within the treated setting. In the interaction models, RS had a statistically significant RS × RT term, meaning the score’s association with survival differs depending on RT exposure. And given the small number of events, the most solid conclusion is that an RT-dependent association exists, rather than focusing too much on the exact subgroup hazard ratio sizes.

One of the main results is that when both axes were modelled together, discrimination improved compared with using either one alone, and both interaction terms stayed significant in the combined model. This supports the idea that microenvironmental hypoxia and intrinsic DDR state capture partly independent sources of radioresponse heterogeneity [7,48]. Hypoxia is driven by things like perfusion, metabolic demand, and angiogenic signalling, while DDR-linked expression reflects how tumour cells regulate checkpoint and repair pathways. So overall, the combined results fit a multi-axis view of radiosensitivity, where both oxygenation context and intrinsic damage-processing capacity help explain why outcomes under RT differ between patients.

External validation in METABRIC gives a useful and much more event-rich check on whether the hypoxia axis generalises. When the fixed three-gene hypoxia score (CP, GPC3, STC1) was applied to METABRIC, it still showed a statistically significant, though smaller, association with overall survival (HR = 1.34, *p* = 0.004), with modest discrimination (C-index ≈ 0.52; 60-month AUC ≈ 0.55). Some attenuation of effect size and significance is expected when transferring a small transcriptomic score across cohorts that differ in expression platform, patient mix, and treatment patterns. Since METABRIC does not report radiotherapy exposure in a way that can cleanly harmonise with the TCGA RT-defined subset, this result should mainly be seen as evidence that the hypoxia score has a reproducible prognostic signal, rather than as an external confirmation of RT-modification effects.

These results could have clinical relevance, but they should be presented as hypothesis-generating rather than practice-changing. Recent reviews have similarly emphasised the growing interest in biomarker-guided radiotherapy, clinical radiosensitivity prediction, and radiosensitisation strategies in breast cancer, which supports the relevance of testing multi-axis molecular models such as the one presented here [16,17]. In principle, a two-axis model could help flag subgroups where standard RT is less effective, or where combination strategies make the most biological sense. For example, patients with high hypoxia or high RS might be candidates to study for altered fractionation, dose intensification, or radiosensitisation approaches [49,50,51,52].

Hypoxia modification (oxygenation strategies or hypoxia/HIF-pathway interventions) and DDR targeting (e.g., ATR/CHK1/PARP pathway inhibitors) both align with the biology these signatures are trying to capture. That said, the therapeutic window and the risk of confounding by baseline tumour aggressiveness would need careful prospective testing before any clinical decisions could be based on a score like this.

We also examined whether these transcriptomic scores were associated with pathologic stage. The hypoxia score showed a significant association with stage in TCGA, whereas the RS score did not. This suggests that some of the hypoxia signal may partly reflect broader tumour aggressiveness or disease extent, rather than being fully independent of stage. Accordingly, the hypoxia-related findings should be interpreted as biologically meaningful but not necessarily stage-independent in this dataset.

Methodologically, this work shows why it helps to combine biological curation with modern modelling approaches. By restricting penalised regression to mechanistically chosen gene pools, we reduce the multiple-testing burden and keep the resulting signatures easier to interpret than if we did an unconstrained genome-wide selection. Also, using interaction models gives a direct statistical test of RT-dependent associations instead of relying only on prognostic effects, which matters when the real aim is to identify potential treatment modification rather than general risk prediction.

There are a few important limitations to keep in mind. The biggest issue is the small number of OS events in TCGA (21 in the 266-patient cohort, and 15 in the 170-patient RT-defined subset). That makes the estimates less precise and can push hazard ratios to look extreme, especially when split into subgroups.

Also, RT information in TCGA is not very detailed. Although the clinical XML allowed extraction of additional RT details, including type, dose, units, fraction number, and timing, these fields were not fully standardised, and some dose values remained heterogeneous or extreme even after unit harmonisation. As a result, the RT metadata were useful for descriptive cohort characterisation but were not sufficiently harmonised for formal dose–response modelling. Since this is a retrospective observational dataset, confounding by indication is also a real concern; RT is not randomly assigned, and it may be linked to stage, subtype, comorbidities, or broader treatment pathways. Finally, overall survival is not the most direct endpoint for RT effect, and outcomes like recurrence or locoregional control were not consistently available.

Histological heterogeneity within breast carcinoma was also not the primary focus of the present modelling framework and should be explored more explicitly in future radiogenomic studies. The analysed cohort consisted of breast carcinoma subtypes rather than the full spectrum of breast malignancies, so the present findings should not be generalised beyond that setting.

Even with these limitations, the fact that the direction of the results is consistent across the different modelling approaches, the Kaplan–Meier splits tell the same story, and both interaction terms stay significant in the combined model all suggest there is a real, biologically meaningful signal here. The next step is to validate this in independent cohorts that have better RT detail and enough events to estimate interactions reliably, ideally with proper multivariable adjustment for clinical factors.

If these findings hold up, combining hypoxia and DDR-linked signatures in one framework could help move toward more biologically driven RT stratification and support the design of biomarker-enriched trials testing radiosensitising combinations. 

## 5. Conclusions

This study highlights two biologically driven transcriptional axes, microenvironmental hypoxia and a DSB-repair-linked radiosensitivity programme, that are informative for overall survival in breast cancer and show evidence of RT-associated effect modification in TCGA. Using mechanistically curated gene pools and interaction-focused modelling, we derived a simple hypoxia score (CP, GPC3, STC1) and an RS score (ATR, RPA2, BLM, MRE11A). In the TCGA RT-defined subset, each axis showed a statistically significant interaction with RT status, and the best discrimination was seen when both axes were modelled together (combined interaction model C-index = 0.785). Overall, this supports the idea that hypoxia and RS capture complementary, non-redundant parts of radiosensitivity-related outcome heterogeneity.

External testing in METABRIC strengthens the generalisability of the hypoxia axis as a prognostic signal. When we applied the fixed three-gene hypoxia score without refitting, it kept a significant, though weaker, association with overall survival in a large independent cohort. METABRIC does not include radiotherapy annotation that can be cleanly matched to the TCGA interaction framework used here, but the result still supports that hypoxia-linked risk ranking transfers across cohorts and expression platforms.

Because this is a retrospective analysis, TCGA has a small number of events, and the RT metadata is not very detailed, these results should be treated as hypothesis-generating when it comes to RT prediction and treatment modification. The key next step is confirmation in independent RT-annotated datasets with harmonised information on dose, timing, and systemic-therapy context. If the findings hold up in prospective settings, a combined hypoxia–radiosensitivity framework could support more biologically guided escalation or de-escalation strategies and also provide a clear rationale for biomarker-guided trials testing hypoxia-targeting and DDR-targeted radiosensitising combinations.

## Figures and Tables

**Table 1:** Availability and descriptive summary of radiotherapy metadata in the RT-defined subset (N = 170).

Characteristic	N	%
RT exposure status available	170	100.0
Radiation type available	150	88.2
Radiation dosage available	141	82.9
Days to RT start available	146	85.9
Days to RT end available	147	86.5
Dose units		
cGy	132	77.6
Gy	13	7.6
Missing dose units	25	14.7
Usable dose and timing available	141	82.9
Timing only available	9	5.3
Neither usable dose nor timing available	20	11.8
Harmonised dose summary (Gy)		
Median dose	60	
Interquartile range	50.4–64	
Fraction number summary		
Median number of fraction	30	
Interquartile range	25–33	

Note: Radiation dosage values were harmonised to Gy where units were available. Because the TCGA radiation fields showed heterogeneous recording and some extreme values, these metadata were used for descriptive cohort characterisation rather than formal dose–response modelling.

**Table 2:** Clinicopathologic characteristics of the TCGA-BRCA overall OS cohort (N = 266).

Characteristic	N	%
Histology		
Infiltrating ductal carcinoma	187	70.3
Infiltrating lobular carcinoma	50	18.8
Other carcinoma	29	10.9
Pathologic stage		
Stage I	27	10.2
Stage IA	16	6.0
Stage IB	2	0.8
Stage II	2	0.8
Stage IIA	90	33.8
Stage IIB	66	24.8
Stage IIIA	41	15.4
Stage IIIB	1	0.4
Stage IIIC	17	6.4
Stage IV	2	0.8
Stage X	2	0.8

## Data Availability

TCGA-BRCA data can be accessed through the Genomic Data Commons (GDC) and FireBrowse portals. The processed expression matrices, clinical annotations, and the full R analysis code will be shared in a public repository GitHub (https://github.com/JimmyCarterOsei/TCGA-BRCA-hypoxia-radiosensitivity, accessed 23 March 2026) once this work is published, or upon reasonable request to the corresponding author.
